# Thrombin promotes fibronectin secretion by bone marrow mesenchymal stem cells via the protease-activated receptor mediated signalling pathways

**DOI:** 10.1186/scrt424

**Published:** 2014-03-17

**Authors:** Jin Chen, Yujie Ma, Zi Wang, Hengxiang Wang, Lisheng Wang, Fengjun Xiao, Hua Wang, Jianming Tan, Zikuan Guo

**Affiliations:** 1 Fujian Provincial Key Laboratory of Transplant Biology, Fuzhou General Hospital, Xiamen University, Fuzhou 350025 China; 2 Department of Experimental Hematology, Institute of Radiation Medicine, Beijing 100850, China; 3 Department of Hematology, Air Force General Hospital, Beijing 100036, China

## Abstract

**Introduction:**

Fibronectin (FN) is commonly used in the development of serum-free media for the expansion of mesenchymal stem cells (MSCs). This study was aimed to observe if thrombin could stimulate FN secretion by human bone marrow MSCs and investigate the potential underlying mechanisms.

**Methods:**

PCR was performed to detect the expression of the protease-activated receptors (PARs) in MSCs. After thrombin treatment, the expression level and secretion of FN were observed by RT-PCR, immunofluorescence staining and ELISA, respectively, and the activation of ERK1/2 and NF kappa B pathways was revealed by Western blotting, with or without pre-treatment of small-molecule blockers specific for PAR-1 and –2. The phenotypic and functional activities of thrombin-treated MSCs were also observed.

**Results:**

PCR analysis showed that human bone marrow MSCs expressed two subtypes of PARs, PAR-1 and PAR-2. Thrombin treatment enhanced MSCs to express FN at mRNA and protein levels and promoted FN secretion by MSCs, accompanied by potent adherence to the culture plastic. Thrombin induced prompt phosphorylation of ERK 1/2 and NF kappa B p65 and the stimulatory effects of thrombin on FN secretion were blunted by specific inhibitors of these signaling molecules. Blockage to PAR-1 and PAR-2 partially abrogated thrombin-elicited FN secretion by MSCs and ERK 1/2 phosphorylation, whereas that of NF kappa B p65 was unaffected. Moreover, thrombin-treated MSCs maintained the phenotypic features, *in vitro* osteogenesis and adipogenesis capacities, and inhibitory activity on Phytohemagglutinin-induced allogeneic lymphocyte proliferation.

**Conclusions:**

Thrombin could promote FN secretion by MSCs via PAR-mediated ERK 1/2 activation, while NF kappa B might be also involved in an undefined manner.

## Introduction

Mesenchymal stem cells (MSCs) are adult multipotent cells that were initially isolated from bone marrow [1] and characterized by the fibroblast-like appearance in culture and the capacities to form bone, adipose and cartilage. Since the first reported clinical trial in 1995, MSCs have been increasingly used for clinical research ranging from immunological intervention to tissue engineering and trauma repair [2-6]. However, the quantity of MSCs is very low in bone marrow (about 0.001 to 0.01% of the mononuclear cells) and *in vitro* expansion is the prerequisite for their clinical application. MSCs can be easily expanded in culture media containing fetal bovine serum (FBS) from selected lots. However, MSCs cultured with this protocol may expose the recipients to the potentially contaminated pathogens in FBS and the risk of sensitization elicited by xenogeneic proteins engulfed into the cytoplasm [7]. Further, the use of FBS from batch to batch in the expansion of MSCs may affect the reproducibility [8].

To overcome these inherent drawbacks of FBS, increasing investigations have been reported to develop animal serum-free and chemically-defined media for MSC expansion. These novel media usually contain human platelet lysates [9-14] or a cocktail of growth factors [15] and extracellular matrix molecules, including fibronectin (FN), collagen and fetuin [15-19] to support MSC proliferation and adhesion to the plastic as well. Generally, MSC expansion with a chemically-defined medium seems to take into consideration the safety and reproducibility in good manufacturing practice conditions [2,20,21]. However, the addition of extracellular matrix in the media is not cost-effective and further investigations are needed to solve this problem.

In fact, MSCs can secrete various cytokines, growth factors and a series of extracellular matrix molecules including collagens and FN [22-26], which are the main substrates for MSC adhesion to the plastic. This phenomenon induced us to search for some stimuli that could promote the secretion of a quantity of matrix molecules by MSCs.

Thrombin is a serine protease which has a variety of biological activities [27]. It can stimulate collagen synthesis in mesangial cells [28,29], and can enhance FN production by human proximal tubular epithelial cells [30]. In this study, the stimulatory effect of thrombin on MSCs was investigated. It was found that thrombin can induce the secretion of FN by MSCs, probably through the protease-activated receptor (PAR) coupling-mediated ERK1/2 pathway, and nuclear factor kappa B (NF-kappa B) signaling might also be involved, though the exact mechanisms need further investigations to be clarified.

## Methods

### Cell culture

This study was approved by the Ethics Review Committee of the Fuzhou General Hospital, and written informed consent was obtained from all participants. Human bone marrow MSCs were cultured and identified as described previously [31,32]. In brief, bone marrow aspirates were obtained from five healthy donors who gave informed consent. Mononuclear cells were isolated by gradient density centrifugation on Ficoll-Paque (1.077 g/ml, GE Healthcare Bio-Sciences AB, Uppsala, Sweden) and suspended in α-Minimum Essential Medium (α-MEM, Gibco Life Technologies, Carlsbad, CA, USA) supplemented with 10% fetal bovine serum (FBS, Hyclone, Beijing, China). The cells were seeded into plastic dishes and non-adherent cells were removed after 48 h. Medium was changed every three days. When the culture reached 80 to 90% of confluence, cells were digested with 0.05% trypsin-EDTA (Gibco Life Technologies, Carlsbad, CA, USA), counted and passaged at a density of 6,000 cells/cm^2^. Cells of passages 3 to 5 were used for the experiments below.

### Standard PCR

MSCs from four donors were harvested and the total cellular RNA was extracted using a total RNA kit II (Omega Bio-Tek, Norcross, GA, USA). The first-strand cDNA was synthesized from 2 μg of total RNA using a RT-PCR kit (Thermo Fermentas, Vilnius, Lithuania) according to the manufacturer’s directions. Semi-quantitative PCR was performed to test the expression PAR subtypes 1 to 4 according to the condition of denaturing at 94°C for 30 sec, annealing at 55°C for 30 sec, and extension at 72°C for 30 sec for 30 cycles. *B-actin* was used as the reference gene. The primers used for PCR are shown in Table 1. The PCR products were separated in a 1% agarose gel and stained with gold view.

**Table 1:** Primer sequences for PCR analyses

**Gene**	**GB. accession**		**Primer sequence (5′-3′)**	**Product size (bp)**
*FN1*	NM_212478.1	Forward	CCCCTGGGGTCACCTATTAC	189
		Reverse	CGGTCAGTCGGTATCCTGTT	
*ACTB*	NM_001101.3	Forward	TGATGATATCGCCGCGCTCGT	168
		Reverse	GCCTCGTCGCCCACATAGGAAT	
*PAR1*	NM_001992.3	Forward	GCCTCCCACTAAACATCATGGC	199
		Reverse	AATGCTGCAGTGACGAAGCG	
*PAR2*	NM_005242.4	Forward	TGTCGTGAAGCAGACCATCTT	167
		Reverse	TCATCAGCACATAGGCAGAGG	
*PAR3*	NM_004101.3	Forward	CTGCTTCTGTTGCCCACTTT	162
		Reverse	AGTAATCGTGGCTCCTGTCC	
*PAR4*	NM_003950.2	Forward	ATGACAGTGACACCCTGGAG	188
		Reverse	GAGGTTCATCAGCAGCATGG	

### Quantitative PCR

Real-time quantitative PCR was performed to quantify FN expression using Agilent brilliant III ultra-fast SYBR green qPCR master mix (Agilent Technologies, Foster, CA, USA) on the ABI 7500 Real-Time PCR System (Applied Biosystems, Carlsbad, CA, USA). Total cellular RNA of MSCs was extracted and cDNA was synthesized as routinely described. The sequences of the primers are shown in Table 1. Relative quantitative determination of FN expression level was performed by comparing the comparative threshold cycle method (ΔCt). The FN expression level was presented as fold change compared with control group (fold change = 2^-ΔΔCt^).

### ELISA

Aliquots of MSCs were seeded into six-well culture plates at a concentration of 1 × 10^5^/well. The cells were allowed to attach to the plastic overnight. The medium was discarded and the cells were washed twice with PBS. Fresh medium without serum was then added and the culture was maintained at 37°C for 24 hours. Graded concentrations of thrombin were added and MSCs were incubated for 24 h, 48 h and 72 h. Also, the cells were exposed to small molecules, including the PAR1 antagonist (SCH79797, 1 μM, Santa Cruz Biotechnology, Santa Cruz, CA, USA), the PAR2 peptide antagonist (FSLLRY-NH_2_, 10 μM, Tocis Bioscience, Bristol, UK), the ERK1/2 inhibitor (PD98059, 20 μM, Sigma-Aldrich, Saint Louis, MO, USA), or the NFκB p65 inhibitor (ethyl pyruvate, 5 mM, Sigma-Aldrich, Saint Louis, MO, USA), for 30 minutes before the cells were treated with thrombin (4 U/ml). The supernatants were collected and the contaminated cell debris was removed by centrifugation at 12,000 g for 10 minutes. The concentration of FN was detected with a commercially available ELISA kit (R&D Systems, Minneapolis, MN, USA) according to the manufacturer’s instructions.

### Western blot

Cells were washed twice with cold PBS, and then lysed with fresh RIPA containing a cocktail of protease inhibitors, including 1 mM PMSF, 5 mM EDTA and 1 mM phosphatase inhibitor. Cells were scraped off the dishes using a cold plastic cell scraper. The cell suspension was transferred into pre-cooled Eppendorf tubes, and maintained at 4°C for 30 minutes with constant agitation. Total protein of the lysates was determined by bicinchoninic acid (BCA) method. Total protein (40 μg/lane) was separated on a 10% SDS gel and then transferred to a polyvinylidene difluoride (PVDF) membrane. Western blot was performed with the following primary antibodies: rabbit monoclonal antibody anti-phospho-NFκB p65, anti-NFκB p65, anti-phospho-ERK 1/2 and anti-ERK 1/2 (Cell Signaling Technology, Danvers, MA, USA, 1:1,000 dilution) in Tris Buffered Saline with Tween (TBST) with 5% bovine serum albumin (BSA). Goat anti-rabbit or anti-mouse antibodies conjugated horseradish peroxidase (Santa Cruz Biotechnology, 1: 2,000 dilution) were used as secondary antibody. The signals were shown with enhanced chemiluminescent reagents (ECL, Thermo Pierce Protein Biology, Rockford, IL, USA).

### Adhesion assay

Thrombin-treated MSCs were washed twice with PBS. Cells (2 × 10^4^) in a volume of 100 μL were added in each well of 96-well plates and kept at 37°C for 1 h for spontaneous adhesion. The non-adherent cells and medium were aspirated, and the wells were washed twice with PBS with vigorous shaking. Aliquots of 100 μl of 3-(4, 5-dimethylthiazol-2-yl)-2, 5-diphenyl-tetrazolium bromide (MTT) solution in alpha-MEM were added at a final concentration of 0.5 mg/ml and incubated at 37°C for 4 h. The formazan was then dissolved in dimethyl sulfoxide (DMSO) and optical density (OD) values were detected at a wavelength of 490 nm.

### Analysis on cell surface markers

MSCs from three individuals were seeded at a density of 6,000 cells/cm^2^ into culture dishes of 100 mm in diameter, allowed to attach overnight and treated with thrombin at a concentration of 4 U/ml for one week. The cells were then collected by trypsin digestion, washed in PBS, and reacted for 30 minutes in the dark with mouse monoclonal antibodies against human CD31, CD34, CD44, CD45, CD73, CD90, CD105, HLA-DR and the corresponding fluorescein-conjugated isotype antibodies. At least 10,000 events per sample were collected with FACScan (BD Biosciences, San Jose, CA, USA). The data were analyzed with FlowJo 7.6 software after the interested events were gated and the negative thresholds were set according to the relative fluorescent intensities of the negative controls.

### Immunofluorescence staining

MSCs were plated in 24-well plates at a density of 1 × 10^4^ cells per well. After stimulated by thrombin for 48 hours, the cells were washed three times with PBS and then fixed with 4% paraformaldehyde (PFA) for 20 minutes. Permeabilization was performed by incubating in PBS containing 0.5% Triton-X for 10 minutes. Unspecific binding sites were blocked with 5% BSA in phosphate-buffered saline with Tween (PBST) for 20 minutes. Cells were incubated with mouse anti-FN antibody (Santa Cruz Biotechnology, sc-271098) or rabbit anti-alpha tubulin antibody (GeneTex, Irvine, CA, USA, GTX102078) at a dilution 1:100 in PBST containing 0.5% BSA overnight at 4°C. After washing in PBS, goat anti-mouse or goat anti-rabbit IgG conjugated FITC was added and incubated for 60 minutes at room temperature. Nuclei were counter-stained with 4',6-diamidino-2-phenylindole (DAPI) for five minutes. The cells were observed and the pictures were taken by a confocal laser scanning microscope (Zeiss LSM510, Carl Zeiss, Oberkochen, Germany).

### *In vitro* differentiation assays

MSCs were suspended in α-MEM containing 1% FBS and cultured in the presence or absence of thrombin at a final concentration of 4 U/ml. The culture was maintained for one week when the adherent confluence reached over 90%. The cells were harvested and re-seeded into six-well culture plates at a density of 30,000 cells/well (for osteogenesis induction) or 90,000 cells/well (for adipogenesis induction). The cells were exposed to the inductive conditions for MSC differentiation into osteoblasts and adipoblasts and the culture was maintained for 10 days as previously reported by our group [33]. Intracellular alkaline phosphotase activity and lipid droplets were revealed by NBT-BCIP or Oil-red O staining, respectively.

### Lymphocyte transformation assay

MSCs or MSCs pretreated with thrombin for one week as described above were seeded into 96-well culture plates at a density of 20,000, 10,000, 4,000, 2,000 or 1,000 cells per well. The cells were allowed to attach overnight and irradiated with a ^60^Co source at a total dose of 30 Gy before the media were aspirated out. Heparinized peripheral blood was collected from three healthy donors and mononulcleated cells were harvested by Ficoll-Hypaque gradient density centrifugation. The mononuclear cells were washed in PBS, suspended in RPMI 1640 with 10% FBS and 5 μg/ml of phytohemagglutinin (PHA) (Sigma-Aldrich, Saint Louis, MO, USA) at a density of 2 × 10^6^ cells/ml, and added into the plates at a volume of 100 μl/well. The cell culture without MSCs alone served as the positive control. The culture was maintained for 72 hours. The MTT test was performed to evaluate the cell viability as described above.

### Statistical analysis

Quantitative data were presented as means ± SE. Statistics were analyzed using SPSS 13.0 software. Multiple group comparisons were performed with one-way ANOVA analysis of variance and comparisons between two groups were completed with Student’s *t-*test. A *P*-value less than 0.05 was considered statistically significant.

## Results

### MSCs express the thrombin PARs

**Figure 1:**
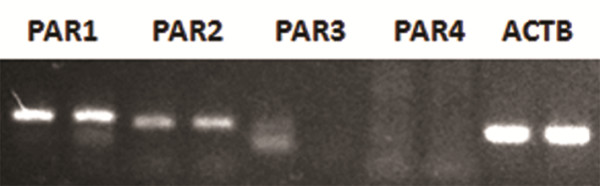
**Expression of thrombin receptors on MSCs.** Semi-quantitative RT-PCR was performed to analyze thrombin receptor (proteinase-activated receptors, PARs) expression on mesenchymal stem cells (MSCs). *Beta-actin* (*ACTB*) was used as the reference gene. MSCs from two donors were analyzed. The results were representative of two individual experiments.

To observe the potential effect of thrombin on MSCs, RT-PCR was first performed to evaluate whether MSC express the receptors for thrombin protease-activated receptors (PARs). The results showed that MSCs expressed PAR-1 and PAR-2, and did not express PAR-3 and PAR-4 (Figure 1), suggesting that thrombin might exhibit its activity through PAR-1 and PAR-2.

### Thrombin enhances expression and secretion of FN by MSCs

**Figure 2:**
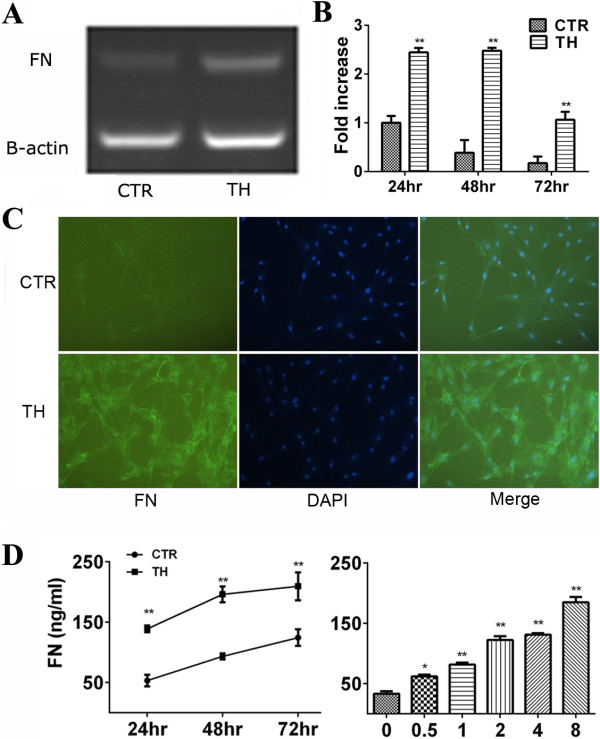
**Thrombin promoted MSCs to express and secrete FN. A-C**: MSCs were serum-deprived for 24 to 48 h and then maintained in the presence or absence (as control, CTR) of thrombin (4 U/ml, TH) for 24 h. FN mRNA expression by MSCs was analyzed by semi-quantitative quantitative RT-PCR **(A)** and real-time quantitative PCR by comparing the ΔCt method. The FN expression level was presented as fold change (fold change = 2^-ΔΔCt^) compared with control group (**B**, **P <0.01). **C**: Cellular immunofluorescence was performed to assess FN expression in BMSCs (Magnification: 100×). **D**: MSCs were treated with thrombin at a dose of 4 U/ml for the indicated times (left) or thrombin at the indicated concentrations for 24 h before the contents of FN in the supernatants were detected by ELISA. The data are representative of those from MSCs from four individual donors. BMSCs, Human bone marrow mesenchymal stem cells; FN, Fibronectin; MSCs, Mesenchymal stem cells.

RT-PCR showed that MSCs expressed FN at a low level at baseline, and its expression was greatly elevated by thrombin treatment (Figure 2A). Quantitative PCR proved that thrombin at a concentration of 4 U/ml augmented FN mRNA expression in a time-dependent manner (Figure 2B). The results were further supported by immunohistology staining with an anti-FN antibody (Figure 2C). Results from ELISA tests showed that the stimulatory effects of thrombin were time- and dose-dependent (Figure 2D). Statistical analysis found that FN concentrations in the thrombin-treated cell supernatants were remarkably enhanced at each time or dose point compared with those from the corresponding controls (*P* <0.01).

### Thrombin promotes MSCs to adhere to the substrate of the culture plate

**Figure 3:**
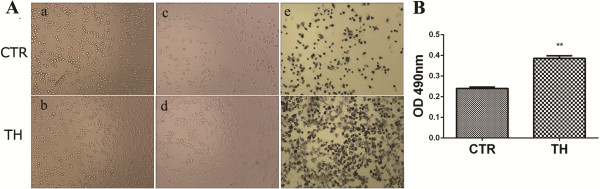
**Thrombin pretreatment promotes the adhesion of MSCs to the culture plastic. A**: Aliquots (2 × 10^4^/100 μl) of MSCs or MSCs were pre-treated with thrombin (4 U/ml) for 24 h were seeded into a 96-well culture plate for spontaneous adhesion for 1 h (a and b), the non-adherent cells was then washed and discarded (c and d). After incubated with MTT at 37°C for three to four hours, the adherent cells were stained by MTT (e and f). **B**: The crystallized formozan was dissolved in DMSO and OD values at a wavelength of 490 nm were detected. CTR: MSCs cultured without thrombin; TH: MSCs treated with thrombin. **P <0.01. The results here are representative of the data from three individual experiments. CTR, Control; DMSO, Dimethyl sulfoxide; MSCs, mesenchymal stem cells; MTT, 3-(4, 5-dimethylthiazol-2-yl)-2, 5-diphenyl-tetrazolium bromide; OD, Optical density.

To further observe if the secreted FN had functional activity, an MTT test was performed to reveal the adhesion of thrombin-treated MSCs to the culture plastic. The results showed that the number of MSCs that had adhered to the culture plate increased markedly after thrombin pretreatment compared with the control group (Figure 3, *P* <0.01). Meanwhile, quantitative RT-PCR showed that thrombin also greatly enhanced the expression of integrin alpha-5 subunit in MSCs. An additional figure shows this result in more detail (see Additional file 1). Therefore, the increased adhesion activity of thrombin-MSCs might not solely be attributed to FN secretion.

### Thrombin activates ERK 1/2 and NFκB pathways

**Figure 4:**
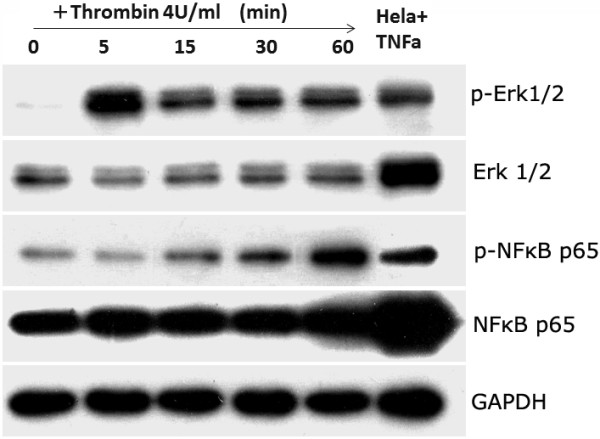
**Thrombin induced the activation of ERK 1/2 and NFκB signalling in MSCs.** MSCs were pre-treated with thrombin (4 U/ml) and collected at the indicated time points for western blotting analysis on the phosphorylation of ERK 1/2 and NFκB p65. The Hela cell line treated with TNF-alpha was used as the positive control. GAPDH was served as the internal reference. The results here are representative of three individual experiments. MSCs, Mesenchymal stem cells; NFκB, Nuclear factor kappa B.

As described above, BMSCs expressed PAR-1 and PAR-2, and the coupling of these two receptors to their ligands has been previously reported to be able to activate ERK 1/2 and NFκB pathways [27]. To observe if this was the case in thrombin-treated MSCs, the activation of these two signaling pathways was tested. The results indicated that treatment of MSCs with thrombin at a concentration of 4 U/mL resulted in a rapid phosphorylation of ERK 1/2, reaching its maximum at 5 minutes post treatment and lasting at least for 60 minutes (Figure 4). Thrombin also induced phosphorylation of NFκB (Figure 4); however, in sharp contrast to ERK 1/2 activation, NFκB p65 phosphorylation was observed at 15 minutes and reached maximum at 60 minutes.

### Inhibition of ERK 1/2 and NFκB p65 pathways partially abrogate thrombin-stimulated FN secretion

**Figure 5:**
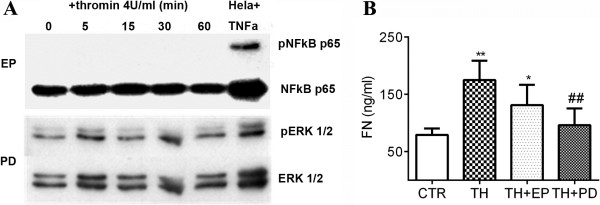
**Inhibitors for NFκB p65 and ERK 1/2 suppress thrombin-stimulated FN secretion. A**: MSCs were pretreated with EP or PD, followed by thrombin treatment with a dose of 4 U/ml. The cells were collected at the indicated time points for Western blotting. **B**: The supernatants were collected after MSCs were cultured for 48 hours in the presence or absence of thrombin (TH, 4 U/ml), EP or PD. The concentrations of FN were assessed by ELISA. **P <0.01 and *P <0.05 compared with control (CTR). ##*P* <0.01 compared with TH. The results were from three individual experiments. EP, Ethyl pyruvate; FN, Fibronectin; MSCs, Mesenchymal stem cells; NFκB, Nuclear factor kappa B; PD, PD98059.

To further determine which pathway took the main effect on FN expression and secretion, the specific inhibitor ethyl pyruvate (EP), which inhibits NFκB signaling by directly targeting p65 subunit [34], and PD98059 (PD), which blocks the ERK 1/2 signaling pathway [35,36], were used separately. Western blotting showed that EP treatment completely inhibited the phosphorylation of NFκB p65 and, PD could greatly down-regulate ERK 1/2 activation (Figure 5A). Concomitantly, EP and PD pretreatment suppressed thrombin-elicited FN secretion by MSCs (Figure 5B). Furthermore, PD seemed to exhibit a more potently inhibitory effect than EP (*P* <0.01), though FN secretion in EP pre-treated MSCs was still significantly increased compared with the serum free medium control group (Figure 5B, *P* <0.05).

### Blockage to PAR suppresses thrombin-stimulated ERK 1/2 activation and FN secretion

**Figure 6:**
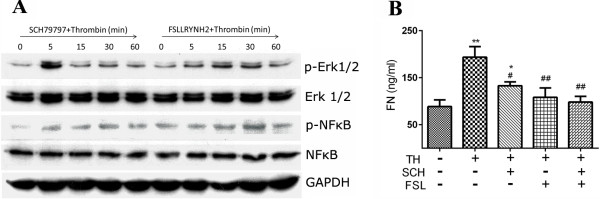
**Blockage to PAR affects the phosphorylation of ERK 1/2 and FN secretion by thrombin-treated MSCs.** SCH79797 (SCH) and FSLLRY-NH_2_ (FSL) were added into MSC culture in the presence of thrombin (TH, 4 U/ml). The cells were harvested at the indicated time points and the phosphorylated status of ERK 1/2 and NFκB p65 was revealed by Western blotting **(A)**. MSC culture was maintained for 48 hours and the supernatants were collected for FN detection by ELISA **(B)**. Data are shown as means ± SE (n = 3). *compared with control group (thrombin- and inhibitors- free), *P <0.05, **P <0.01; # compared with thrombin 4 U/ml treated group, #*P* <0.05, ##*P* <0.01. FN, Fibronectin; MSCs, Mesenchymal stem cells; NFκB, Nuclear factor kappa B.

To observe how thrombin elicited NFκBp65 and ERK 1/2 activation, PAR-1 specific antagonist SCH79797 [37-39] and PAR-2 specific blockade agent FSLLRY-NH_2_[40-42] were added in the culture of thrombin-treated MSCs, and the activation status of NFκB p65 and ERK 1/2 was revisited. The results showed that after treated by SCH79797 (1 μM), the early phosphorylation of ERK 1/2 (at the time-point of five minutes) was not inhibited while its continuous activation was suppressed (Figure 6A). In FSLLRY-NH_2_ (10 μM) -treated MSCs, the phosphorylation of ERK 1/2 was greatly inhibited. However, blockage to PAR-1 and PAR-2 had little effect on the phosphorylation status of NFκB p65 when they were used respectively. Furthermore, the effects of thrombin on the FN secretion of MSCs was significantly inhibited (*P* <0.01) when the PAR-1 or PAR-2 antagonists were added. The inhibitory effect was greatly obvious when both of them were employed (Figure 6B). The results suggest that PAR signaling is involved in thrombin-elicited FN secretion, at least partially through ERK1/2 pathway.

### Multiple features of thrombin-treated MSCs

**Figure 7:**
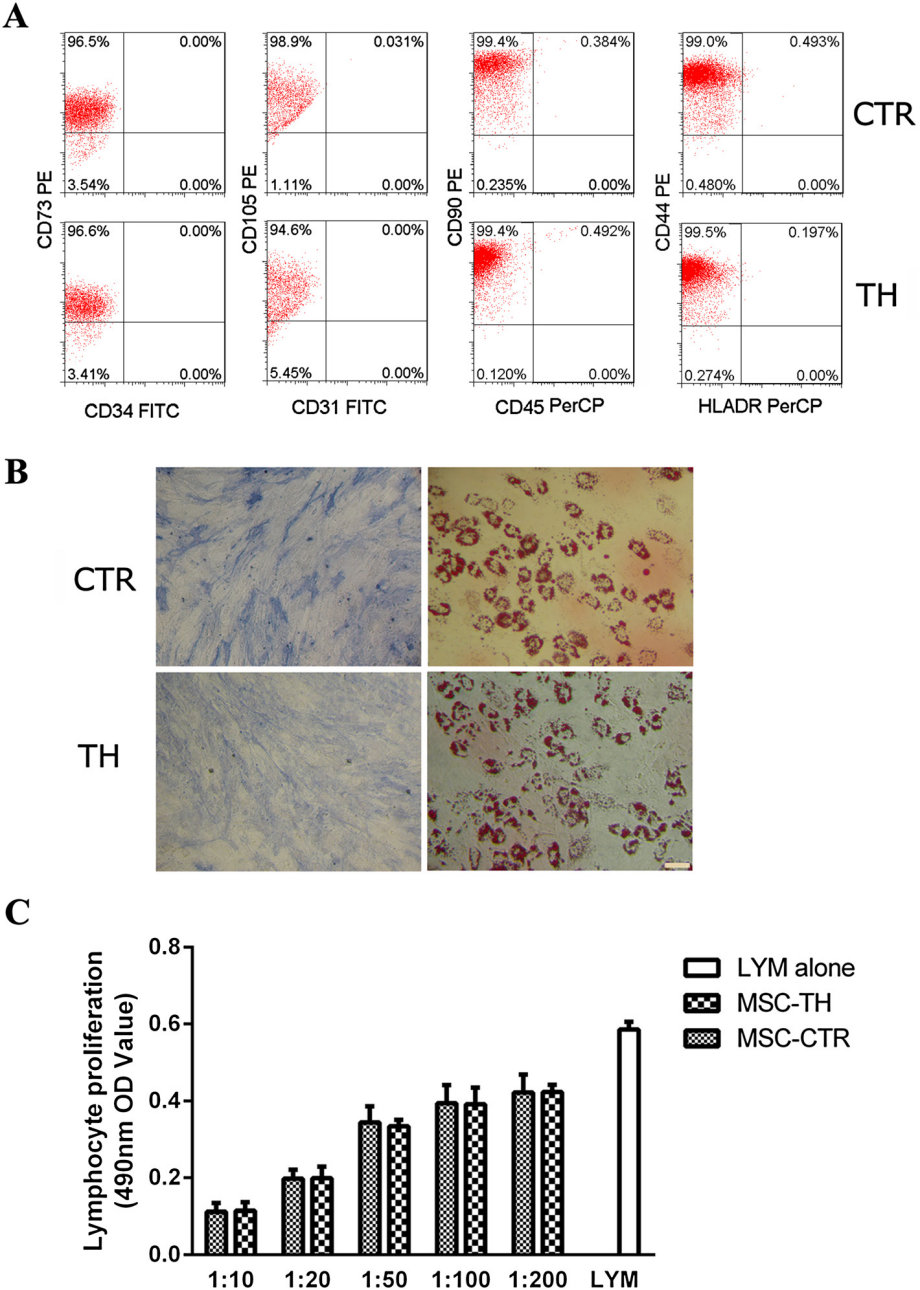
**Thrombin could not change phenotypic and functional features of MSCs.** Human bone marrow MSCs were cultured in the presence of thrombin (4 U/ml) for one week, followed by cell collection for phenotypic and functional analysis. **A**: Flow cytometry analysis on the surface markers. The positivity of the indicated antigens on the parent and thrombin-treated MSCs is provided. **B**: Alkaline phosphatase and Oil-red O staining after the parent and thrombin-treated MSCs were induced for differentiation under the specific conditions. Bar: 50 μm. **C**: Graded doses of MSCs, treated with or without thrombin, were seeded into 96-well culture plate, irradiated and co-cultured for 72 hours with allogeneic peripheral blood mononuclear cells in the presence of PHA. MTT assay was used to reveal the viable lymphocytes. X-axis: the number ratios of MSCs versus mononuclear cells are provided. Lymphocyte: cells cultured in the presence of PHA and absence of MSCs. The results are representative of three individual experiments. MSCs, Mesenchymal stem cells; MTT, 3-(4, 5-dimethylthiazol-2-yl)-2, 5-diphenyl-tetrazolium bromide; PHA, Phytohemagglutinin.

Thrombin is a pleiotropic molecule and thrombin treatment might affect the biological features of MSCs. To observe if it were the case, MSCs were cultured in the presence of thrombin for a week. The cells were then harvested and the surface marker profile, *in vitro* osteogenesis and adipogenesis, and the inhibitory activity on PHA-stimulated proliferation of allogeneic lymphocytes were analyzed. Flow cytometry showed that thrombin-treated MSCs were homogenously positive for CD44, CD73, CD90 and CD105 and negative for CD31, CD34, CD45 and HLA-DR (Figure 7A). Further, they could be induced into osteoblasts and adipoblasts under the standard conditions (Figure 7B). The results suggested that thrombin-treated MSCs met the generally accepted features of human MSCs in culture [43]. Moreover, a MTT test showed that the treated cells, similar to the parent MSCs, could inhibit lymphocyte proliferation elicited by PHA (Figure 7C). The cellular microtube structure revealed by alpha-tubulin staining seemed unaffected by thrombin. An additional figure file shows this in more detail (see Additional file 2).

## Discussion

FN has a variety of biological activities involved in cell adhesion, migration, cytoskeletal organization, proliferation and differentiation [44]. Thrombin has various biological effects in addition to its role in hemostasis and cytoskeletal reorganization, including the secretion of chemokine and the synthesis of matrix metalloproteinases growth factors [45-47]. Here we have demonstrated that thrombin can promote MSC expression and secretion of FN under serum free condition. FN secretion of MSCs was increased nearly three-fold by being stimulated with thrombin. Furthermore, thrombin could improve MSC attachment to the plastic, and have little effect on the phenotypic, differentiation and immunomodulatory features of MSCs. As exogenous FN has been commonly used in commercially available media for MSC expansion, the results here suggest the potential application of thrombin in the development of serum-free culture media [2].

Thrombin exerts many of its actions through PARs [48,49]. PARs have four subtypes, PAR-1, PAR-2, PAR-3 and PAR-4 [48]. We have found that MSCs express thrombin receptors PAR-1 and PAR-2 as evidenced by RT-PCR. It was reported that PAR-mediated signaling via G proteins activates multiple downstream pathways in other type of cells [50]. In the report here, we demonstrated that ERK 1/2 and NFκB p65 signaling pathways in MSCs were activated after treatment with thrombin. The activated NFκB p65 subunit is usually accumulated in the nuclei, recruiting nuclear snail1 and binding to the FN1 promoter, resulting in the activation of fibronectin transcription [51]. In addition, ERK 1/2 activation has been reported to take part in the fibronectin secretion by human dermal fibroblasts [52] and rat mesangial cells [53]. Furthermore, the promoting effects of thrombin on FN secretion observed in this study were reduced by pretreatment with ERK 1/2 inhibitor (PD98059) and NFκB p65 inhibitor (ethyl pyruvate). The reduction was more evident when ERK 1/2 pathway was inactivated. The results suggest that thrombin induces FN secretion of MSCs mainly via ERK 1/2 pathway and activation of NFκB p65 signaling might also be involved.

To further investigate the potential mechanisms underlying the observation that thrombin induced FN secretion, MSCs were pretreated with the PAR-1 signaling antagonist SCH79797 and the PAR-2 peptide antagonist FSLLRY-NH_2_, the activation of ERK 1/2 and NFκB p65 and subsequent enhanced FN secretion by MSCs were then investigated. Our data showed that blockage to PAR-1 could not down-regulate thrombin-stimulated ERK 1/2 phosphorylation at the time-point of five minutes, though the activation of ERK 1/2 was inhibited thereafter. Meanwhile, blockage to PAR2 greatly blunted ERK 1/2 activation at any indicated time points. The down-regulation of the ERK pathway was accompanied by subsequent depression of FN secretion stimulated by thrombin. The results might imply that both PAR-1 and PAR-2 are involved in this process. In endothelial cells, PAR-1 has been found to play a central role in thrombin-induced induction of the ERK-pathway [54], while PAR-2 has been regarded as the indispensable regulator in thrombin-induced expression of decay-accelerating factor, in which the ERK pathway was also involved [50]. This inconsistency could be explained by the observations that PAR-1 and PAR-2 could form a heterodimer and this complex could be activated by thrombin to arouse the phosphorylation of ERK1/2 [55]. Therefore, it is plausible to postulate that thrombin stimulates MSCs to express and secrete FN via PAR-1- and PAR-2-mediated ERK pathway. Interestingly, blockage to PAR-1 and PAR-2 had little effect on the phosphorylation of NFκB, though PAR-1- and PAR-2-mediated NFκB activation by thrombin has been reported in epithelial and endothelial cells [56,57]. The discrepancy should be due to different cell types used in the experiments, and further investigations are needed to clarify the observation that thrombin resulted in NFκB activation in human bone marrow MSCs.

Thrombin is a potent regulator for the functionality of many kinds of cells. In the present study, it is found that thrombin could enhance the secretion of FN by human MSCs, and thrombin-treated MSCs maintain their unique surface marker profile, cellular microtube structural organization, differentiation capacity and immunoregulatory activity. The results suggest that the features of MSCs are not significantly changed after thrombin treatment, and the data are indicative of the applicable potential of thrombin in the development of serum-free media for human MSC expansion. However, further experiments should be performed to evaluate the safety and function of thrombin-treated MSCs after serial passaging before thrombin is used in the expansion medium in the clinical setting.

## Conclusions

In conclusion, thrombin can stimulate human bone marrow MSCs to secrete FN probably through PAR-1- and PAR-2-mediated ERK signaling, though NFκB p65 might also be involved. Further detailed investigations on the properties of thrombin-treated MSCs should be performed to identify if thrombin is used in MSC preparation in clinical trials.

## Abbreviations

α-MEM: Minimum Essential Medium alpha; BCIP: 5-Bromo-4-Chloro-3-Indolyl Phosphate; BMSCs: Human bone marrow mesenchymal stem cells; BSA: Bovine serum albumin; ECL: Enhanced chemiluminescence; EP: Ethyl pyruvate; ERK: Extracellular regulated protein kinases; FBS: Fetal bovine serum; FN: Fibronectin; MSCs: Mesenchymal stem cells; MTT: 3-(4, 5-dimethylthiazol-2-yl)-2, 5-diphenyl-tetrazolium bromide; NBT: Nitro blue tetrazolium; PAR: Protease-activated receptor; PBS: Phosphate-buffered saline; PD: PD98059; PHA: Phytohemagglutinin; QPCR: Real time quantitive polymerase chain reaction; RT-PCR: Reverse transcription polymerase chain reaction; TH: Thrombin.

## Competing interests

The authors declare that they have no competing interests.

## Authors' contributions

JC contributed to the conception and design of the study, data collection and analysis, manuscript preparation and final approval of the manuscript. YJM contributed to data collection and analysis and final approval of the manuscript. HW and FJX participated in data analysis and final approval of the manuscript. ZW, HXW and JMT participated in data collection and final approval of the manuscript. LSW contributed to data analysis, manuscript preparation and final approval of this manuscript. ZKG contributed to conception and design, data analysis, financial support, manuscript preparation and final approval of the manuscript. All authors read and approved the final manuscript.

## Supplementary Material

Additional file 1 **Thrombin up-regulates integrin alpha-5 (ITGA5) in human bone marrow MSCs.** MSCs were treated by thrombin (4 U/ml) for 24 h and quantitative RT-PCR was performed to detect the expression level of ITGA5.The primer sequences were as follows: Forward 5′-GAAGCAGAAGGGAGGGGTAC-3′; Reverse 5′-GGGGTCCAAGGAGAAGTTGA-3′. The results showed that thrombin could significantly enhance MSCs to express ITGA5 (*P* = 0.045, n = 4). Click here for file

Additional file 2 **The cellular microtube structure revealed by alpha-tubulin staining.** MSCs were cultured in the absence (left) or in the presence (right) of thrombin (4 U/ml) for 72 h. The cells were fixed, treated with 0.5% Triton X 100, and were incubated with rabbit antibody against human alpha tubulin at a dilution 1:100 overnight at 4°C. After washing in PBS, goat anti-rabbit IgG conjugated FITC was added and incubated for 60 minutes at room temperature. Nuclei were counter-stained with DAPI for 5 minutes. The cells were observed under a confocal laser scanning microscope (Zeiss LSM510, Carl Zeiss, Oberkochen, Germany). Bar: 10 μm. The figures are representative of two individual experiments. Click here for file
