# Supplementary figures and images for: Thrombin promotes fibronectin secretion by bone marrow mesenchymal stem cells via the protease-activated receptor mediated signalling pathways
Source: Stem Cell Res Ther. 2014 Mar 17;5(2):36. doi: 10.1186/scrt424 (PMC4055141; doi:10.1186/scrt424)

Additional files 1

**
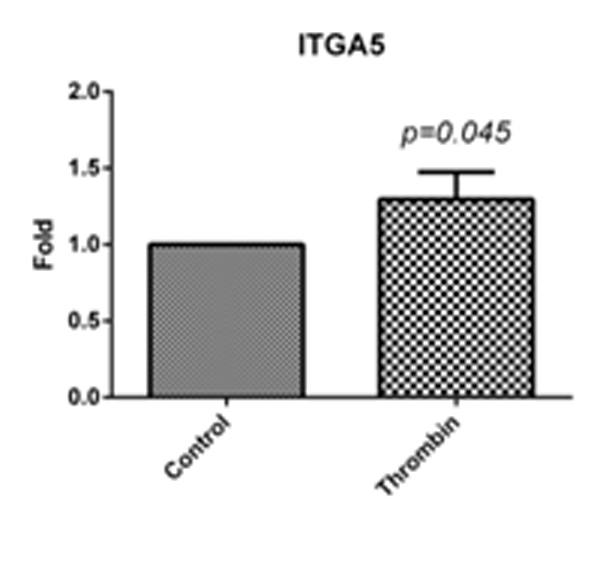
**

Supplement: Additional file 1 — Thrombin up-regulates integrin alpha-5 (ITGA5) in human bone marrow MSCs. MSCs were treated by thrombin (4 U/ml) for 24 h and quantitative RT-PCR was performed to detect the expression level of ITGA5.The primer sequences were as follows: Forward 5′-GAAGCAGAAGGGAGGGGTAC-3′; Reverse 5′-GGGGTCCAAGGAGAAGTTGA-3′. The results showed that thrombin could significantly enhance MSCs to express ITGA5 (P = 0.045, n = 4). [file scrt424-S1.doc]

Additional file 2


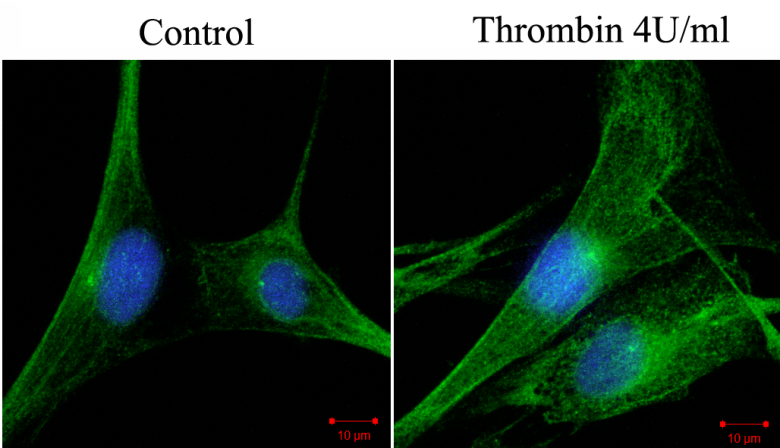

Supplement: Additional file 2 — The cellular microtube structure revealed by alpha-tubulin staining. MSCs were cultured in the absence (left) or in the presence (right) of thrombin (4 U/ml) for 72 h. The cells were fixed, treated with 0.5% Triton X 100, and were incubated with rabbit antibody against human alpha tubulin at a dilution 1:100 overnight at 4°C. After washing in PBS, goat anti-rabbit IgG conjugated FITC was added and incubated for 60 minutes at room temperature. Nuclei were counter-stained with DAPI for 5 minutes. The cells were observed under a confocal laser scanning microscope (Zeiss LSM510, Carl Zeiss, Oberkochen, Germany). Bar: 10 μm. The figures are representative of two individual experiments. [file scrt424-S2.doc]
